# Epigenetic Biomarker to Support Classification into Pluripotent and Non-Pluripotent Cells

**DOI:** 10.1038/srep08973

**Published:** 2015-03-10

**Authors:** Michael Lenz, Roman Goetzke, Arne Schenk, Claudia Schubert, Jürgen Veeck, Hatim Hemeda, Steffen Koschmieder, Martin Zenke, Andreas Schuppert, Wolfgang Wagner

**Affiliations:** 1 Joint Research Center for Computational Biomedicine, RWTH Aachen University, Aachen, Germany; 2 Aachen Institute for Advanced Study in Computational Engineering Science (AICES), RWTH Aachen University, Aachen, Germany; 3 Institute for Biomedical Engineering – Cell Biology, RWTH Aachen University Medical School, Aachen, Germany; 4 Helmholtz-Institute for Biomedical Engineering, RWTH Aachen University Medical School, Aachen, Germany; 5 Bayer Technology Services GmbH, Leverkusen, Germany; 6 Department of Hematology, Oncology, Hemostaseology and Stem Cell Transplantation, RWTH Aachen University Medical School, Aachen, Germany; 7 Institute of Pathology, RWTH Aachen University Medical School, Aachen, Germany

## Abstract

Quality control of human induced pluripotent stem cells (iPSCs) can be performed by several methods. These methods are usually relatively labor-intensive, difficult to standardize, or they do not facilitate reliable quantification. Here, we describe a biomarker to distinguish between pluripotent and non-pluripotent cells based on DNA methylation (DNAm) levels at only three specific CpG sites. Two of these CpG sites were selected by their discriminatory power in 258 DNAm profiles – they were either methylated in pluripotent or non-pluripotent cells. The difference between these two β-values provides an Epi-Pluri-Score that was validated on independent DNAm-datasets (264 pluripotent and 1,951 non-pluripotent samples) with 99.9% specificity and 98.9% sensitivity. This score was complemented by a third CpG within the gene *POU5F1* (OCT4), which better demarcates early differentiation events. We established pyrosequencing assays for the three relevant CpG sites and thereby correctly classified DNA of 12 pluripotent cell lines and 31 non-pluripotent cell lines. Furthermore, DNAm changes at these three CpGs were tracked in the course of differentiation of iPSCs towards mesenchymal stromal cells. The Epi-Pluri-Score does not give information on lineage-specific differentiation potential, but it provides a simple, reliable, and robust biomarker to support high-throughput classification into either pluripotent or non-pluripotent cells.

The possibility of reprogramming somatic cells into induced pluripotent stem cells (iPSCs) has revolutionized stem cell research^1^. However, only a fraction of cells are successfully transformed into pluripotent state by current reprogramming strategies and most of the cells remain non-pluripotent or partially reprogrammed^2^,^3^. Pluripotent stem cells are capable to differentiate into any somatic cell of the human body, whereas incompletely or partially reprogrammed cells may even form cells of all three germ layers but do not exhibit all the characteristics of completely pluripotent cells – thus, by definition, pluripotency can only be proven by adequate *in vivo* assays^4^,^5^. On the other hand, tetraploid complementation or chimera formation, which have been established for the murine system, are not applicable for human iPSCs. Therefore, alternative methods are usually applied to classify cell preparations more vaguely into pluripotent and non-pluripotent cells: The teratoma assay is often considered as gold standard for pluripotency testing of human iPSCs, but it was recently criticized for ethical concerns and lack of standardization^6^. Furthermore, functional markers – such as *in vitro* differentiation assays towards all three germ layers – are relatively time- and labor-intensive. Staining of molecular markers (e.g. OCT4, NANOG, TRA-1-60) via immunofluorescence imaging or flow cytometry are routinely performed, but do not provide quantitative information^7^,^8^. Expression of pluripotency-associated genes can be assessed by quantitative RT-PCR^9^,^10^. However, gene expression is highly dependent on cell growth and requires cutoffs that are not easy to standardize. Alternatively, it is possible to use more complex bioinformatics assays based on whole genome gene expression profiles. For example, PluriTest has proven to be a robust and highly standardized animal free alternative to the teratoma assay^11^, but the required microarray profiles are still relatively expensive for high-throughput analysis of individual clones. Thus, there is always a trade-off between cost- or time-intensiveness and reliability. Furthermore, most of the above mentioned methods cannot distinguish between ESCs or iPSCs, and embryonal carcinomas or parthenogenic ESCs.

Cellular differentiation is reflected by the epigenetic makeup. The DNA-methylation (DNAm) levels at individual CpGs – usually referred to as beta-values (β-values) – can vary continuously between non-methylated (0% DNAm) and methylated (100% DNAm). It has been shown that pluripotent cells have a unique and characteristic epigenetic signature that reflects their broad developmental potential^12^. Hence, analysis of β-values may provide a good measure for molecular definition of iPSCs. Many groups have demonstrated that DNAm profiles of pluripotent stem cells differ considerably in comparison to other cell types^13^,^14^,^15^. iPSCs converge to a characteristic ground state that closely resembles that of embryonic stem cells (ESCs)^16^,^17^,^18^, although it has also been shown that iPSCs retain a residual ‘epigenetic memory' of their tissue of origin^18^,^19^. Within the last years, many well-curated datasets on iPSCs and ESCs have been deposited in public data repositories, which provide new opportunities for identification of epigenetic biomarkers^20^. In this study, we have systematically compared DNAm profiles of cells that were either classified by the authors as pluripotent (ESCs and iPSCs) or non-pluripotent cells to select specific CpG sites that facilitate best discrimination. We hypothesized that two CpGs might be sufficient for a reliable classification: One CpG-site that is typically methylated, and one that is non-methylated in pluripotent stem cells. This Epi-Pluri-Score correctly classified many cell types and cell lines.

## Results

### Derivation of an Epigenetic Pluripotency Marker

**Figure 1:**
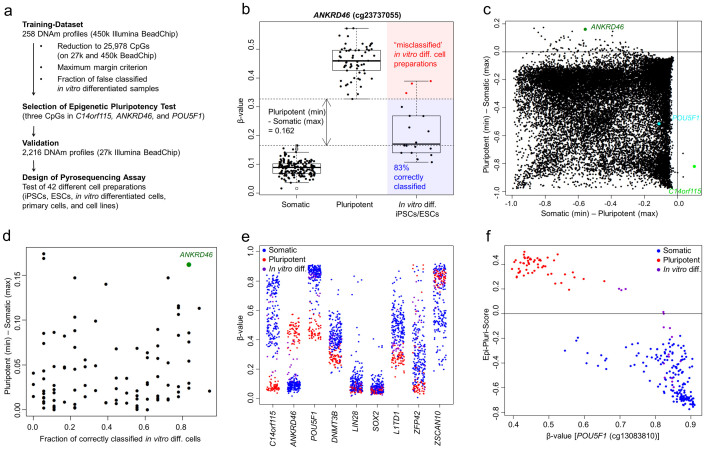
Derivation of the Epi-Pluri-Score. (a) Schematic overview of the work flow. (b) Illustration of the two criteria used for identification of relevant CpG-sites is exemplarily depicted for a CpG site in *ANKRD46*: the margin between pluripotent and somatic samples (arrows; first criterion) and the fraction of correctly classified *in vitro* differentiated iPSCs (second criterion). (c) Margins between the ranges of pluripotent and somatic cells (negative values correspond to overlapping ranges) for all investigated CpG-sites. (d) Plot of the two criteria, which led to the selection of the CpG-site within *ANKRD46*. (e) DNAm levels of the two CpGs of the Epi-Pluri-Score (*C14orf115* and *ANKRD46*) and various pluripotency-associated genes in 258 samples of the training-dataset. (f) Classification of samples of the training-dataset according to Epi-Pluri-Score and DNAm level at cg13083810 (*POU5F1*).

As training-dataset we used DNAm profiles that were generated on the Illumina HumanMethylation450 BeadChip, addressing 485,577 CpG dinucleotides at a single-nucleotide resolution^21^,^22^. 258 DNAm profiles from the Gene Expression Omnibus (GEO) database were curated and rigorously classified into pluripotent cells (63 samples), somatic cells (177 samples), and pluripotent cells upon *in vitro* differentiation (18 samples; Supplemental Table S1). To facilitate subsequent comparison with the many available datasets generated with the Illumina HumanMethylation27 BeadChip, we focused specifically on 25,978 CpGs that are present on both platforms (Figure 1a). For each of these CpGs we compared the ranges of β-values in pluripotent and somatic cells and selected those, which revealed maximum difference between the lowest β-value of the pluripotent cells and the highest β-value of the somatic cells, or *vice versa* (Figure 1b, c). Based on this criterion, we identified the CpG-site cg22247240 that corresponds to the chromosome 14 open reading frame 115 (*C14orf115*, also known as *vertnin*; *VRTN*). This CpG consistently revealed a lower DNAm level in each of the pluripotent samples as compared to each of the non-pluripotent samples. However, for the opposite case (i.e. consistently higher DNAm in pluripotent stem cells as compared to somatic cells) several CpG-sites performed similarly well. Therefore, we utilized a second criterion based on 18 DNAm profiles of pluripotent cells upon *in vitro* differentiation (Supplemental Table S1)^14^,^23^. Overall, the DNAm patterns of these *in vitro* differentiated samples still closely resembled those of pluripotent cells – particularly in three samples that underwent only three days of spontaneous differentiation (GSE30654). The CpG site cg23737055, localized within the gene of ankyrin repeat domain-containing protein 46 (*ANKRD46*), correctly classified 83% of the *in vitro* differentiated samples into the non-pluripotent category (all except of the three days spontaneously differentiated cells) and was therefore chosen for subsequent analysis (Figure 1b–d). The DNAm levels of the two CpGs selected were linearly combined into an Epigenetic Pluripotency Score (Epi-Pluri-Score = β-value [cg23737055] − β-value [cg22247240]), which ranges between 1 and −1. Positive values would be assigned as pluripotent and negative values as non-pluripotent.

Overall, DNAm is rather associated with down-regulation of gene expression – particularly for CpGs localized in promoter regions^24^ – and hence CpGs in pluripotency genes might also provide suitable biomarkers for pluripotency. Therefore, we alternatively selected the CpG site with the highest discriminatory power between pluripotent and somatic cells in several pluripotency-associated genes (e.g. *POU5F1*, *DNMT3B*, *LIN28*, *SOX2*, *L1TD1*, *ZFP42*, and *ZSCAN10*). As expected, the discriminatory power of the above identified two CpGs (associated with *C14orf115* and *ANKRD46*) was higher than any CpG in these pluripotency-associated genes (Figure 1e). However, when focusing on the *in vitro* differentiated samples, it appeared that the CpG site cg13083810 – that is associated with the pluripotency master regulator gene *POU5F1* (octamer binding transcription factor 4, OCT4)^25^,^26^ – was particularly well suited to discern early differentiation events. Therefore, we decided to complement the Epi-Pluri-Score with the β-value at the CpG site cg13083810 (*POU5F1*; Figure 1f). Subsequently, we analyzed the discriminatory power of neighboring CpGs in the genome that are represented by the Illumina HumanMethylation450 BeadChip: Pluripotency-associated methylation patterns were restricted to relatively small regions in *C14orf115* and *ANKRD46*. In contrast, *POU5F1* revealed several neighboring CpGs with similar discriminatory power (Supplemental Fig. S1).

### Validation of the Epi-Pluri-Score in independent datasets

**Figure 2:**
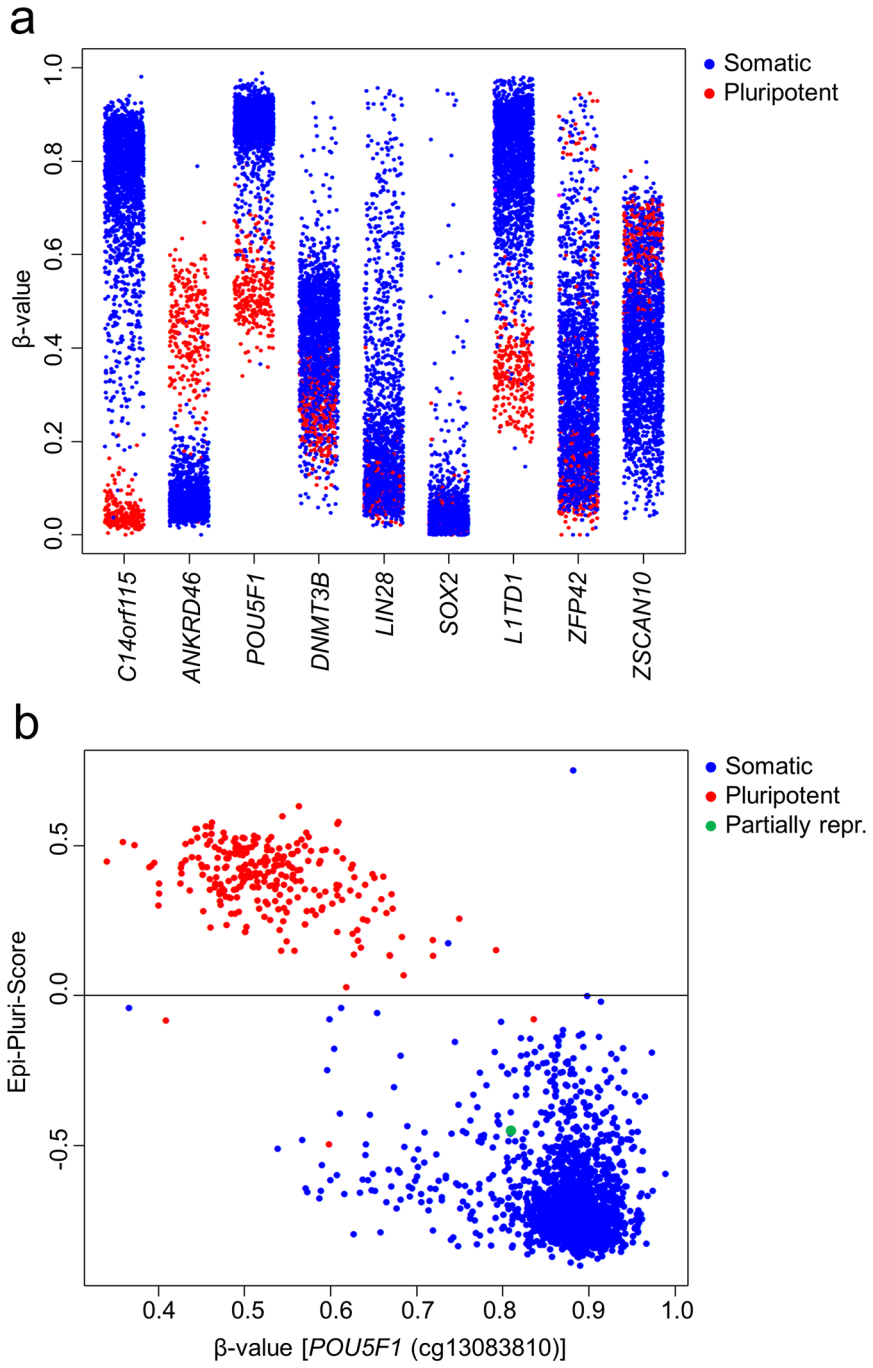
Validation of the Epi-Pluri-Score. (a) DNAm levels at selected CpG sites in samples of the validation-dataset (264 pluripotent and 1,951 non-pluripotent samples; all analyzed with the Illumina HumanMethylation27 BeadChip). The best performing CpG site of the corresponding gene is depicted and CpGs of the Epi-Pluri-Score (*C14orf115*, *ANKRD46*) facilitate best classification. (b) Classification of the validation-dataset using the Epi-Pluri-Score and DNAm of cg13083810 (*POU5F1*). There are only 2 false positive and 3 false negative classifications. Annotation of a partially reprogrammed cell line (green) was later corrected.

To validate the Epi-Pluri-Score we compiled a second dataset consisting of 264 pluripotent and 1,951 non-pluripotent samples that have been analyzed on the Illumina HumanMethylation27 BeadChip platform (Supplemental Table S2). In analogy to the training-dataset the DNAm level at the two CpG-sites within *C14orf115* and *ANKRD46* could clearly separate pluripotent and somatic cells, with few exceptions (Figure 2a). Furthermore, they were amongst those with the highest accuracy to separate pluripotent and somatic cells (Supplemental Fig. S2). Only three samples (GSM744696, GSM755489, and GSM755490) out of 264 pluripotent samples were falsely classified as non-pluripotent by the Epi-Pluri-Score and only two (GSM813270, and GSM615043) out of 1,951 somatic samples were misclassified as pluripotent (Figure 2b). Thus, it has an overall specificity of 99.9% and a sensitivity of 98.9% in the validation-dataset. It might be anticipated that the accuracy could be increased using all CpGs that perfectly separate the two groups in the training-dataset, i.e. all CpGs that are above the horizontal line or to the right of the vertical line in Fig. 1c. In order to stick to the concept of margin maximization, we used a support vector machine with linear kernel to develop a corresponding classifier based on 68 CpGs. Notably, combination of these additional targets did not further increase the accuracy (four pluripotent and three somatic samples were misclassified), indicating that the relatively simple Epi-Pluri-Score provides already quite optimized discrimination.

Subsequently, we focused on the misclassified samples: One of the false positive results (GSM615043) corresponds to ESC-derived hepatocytes that are also close to pluripotent cells in PluriTest analysis of corresponding gene expression profiles^11^ (Supplemental Fig. S3b). An iPSC line, which was initially identified as false negative result turned out to be partially reprogrammed (OCT4 positive, TRA-1-60 negative, refractory to differentiation induction *in vitro* and *in vivo*)^27^ (Figure 2b). One dataset was removed from the validation-dataset after careful reevaluation (GSE35912)^28^: This study investigated iPSCs derived from lung cancer cells that were “misclassified” as non-pluripotent, whereas ESCs from this study were correctly classified as pluripotent (Supplemental Fig. S3a). However, further analysis of the corresponding gene expression profiles, including PluriTest analysis, indicated that the iPSCs were not properly reprogrammed (Supplemental Fig. S3b–d). Furthermore, we could identify a dataset, which appeared to be misarranged in the GEO database (GSE40909, i.e. the order of sample annotations did not match the order of the data themselves)^23^ – upon notification of the corresponding author this error has been corrected. These results exemplify, that the Epi-Pluri-Score is a valuable tool to assist classification of DNAm profiles into pluripotent and non-pluripotent samples.

To further analyze whether the Epi-Pluri-Score can also discern other cell types with high similarity to ESCs or iPSCs - such as embryonal carcinomas (ECs) or parthenogenic ESCs (pESCs) - we subsequently included DNAm profiles of three EC-lines (Tera2, NT2/D1-R1, and 2102Ep) and six pESC-lines to the validation-dataset. All of these abnormal cells were classified as pluripotent by the Epi-Pluri-Score (Supplemental Fig. S4a). Interestingly, EC samples differed in DNAm of the *POU5F1*-related CpG (cg13083810), being either hypo- or hypermethylated – however, this finding needs to be validated in larger datasets. Furthermore, it has been shown that DNAm of imprinted genes is different in pESCs compared to normal ESCs^29^. We therefore reasoned that addition of further CpGs might discriminate between these two cell types. For the six pESC lines additional HumanMethylation450 DNAm profiles were available for the training-dataset. In analogy to the above mentioned 1^st^ criterion we compared ranges of β-values of CpGs and selected those, which revealed maximum difference between ESCs/iPSCs and pESCs (Supplemental Fig. S4b). This approach discerned two CpGs that are associated with the genes for SNRPN upstream reading frame protein (*SNURF*) and the long non-coding RNA *H19*, which are both in fact related to imprinting. Combination of these two CpGs correctly classified the same samples in HumanMethylation27 BeadChip data (Supplemental Fig. S4c, d)^14^. However, the reliability of this “Parthenogenic-Score” needs to be further validated on much larger datasets that are not available at the time.

### Pyrosequencing analysis of the Epi-Pluri-Score

**Figure 3:**
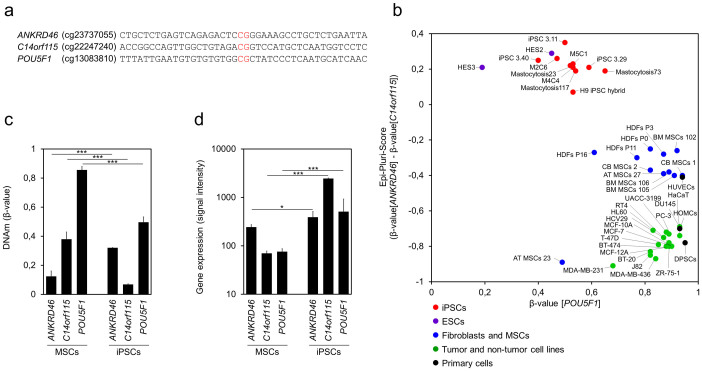
Epi-Pluri-Score analysis by pyrosequencing of selected CpGs. (a) Pyrosequencing assays were designed for the three relevant CpGs (indicated in red). (b) Using this method, various different cell types were analyzed (including ESCs, iPSCs, MSCs, HDFs, tumor cell lines, and other primary cells; Supplemental Table S3). All of the analyzed samples were correctly classified by the Epi-Pluri-Score. (c) DNAm levels and (d) gene expression levels (both based on microarray data) of the genes *ANKRD46*, *C14orf115* and *POU5F1* in iPSCs and MSCs indicate that hypomethylation in *C14orf115* and *POU5F1* may be associated with up-regulation of gene expression (* = P < 0.05; ** = P < 0.02; *** = P < 0.001).

We established pyrosequencing assays to specifically address DNAm levels at the three relevant CpG sites without need of genome wide DNAm profiling (Figure 3a; and Suppl. Fig. S5). These assays were used on DNA of two ESC lines (HES2 and HES3), ten iPSC clones, four preparations of human dermal fibroblasts (HDFs), seven preparations of mesenchymal stromal cells (MSCs), primary cells of three different tissues, and seventeen different established cell lines of various cell types (Supplemental Table S3). Based on pyrosequencing analysis, the Epi-Pluri-Score could successfully classify all pluripotent and non-pluripotent cell preparations (Figure 3b). In analogy to the Illumina HumanMethylation BeadChip data, the CpG site within *POU5F1* was less suitable to discriminate pluripotent and non-pluripotent cells.

### Further analysis with the Epi-Pluri-Score

To determine whether or not the DNAm changes within the genes *C14orf115*, *ANKRD46*, and *POU5F1* are associated with corresponding gene expression changes we exemplarily analyzed gene expression profiles of MSCs and iPSCs^18^,^30^. Hypermethylation of the CpGs within *C14orf115* and *POU5F1* seems to be associated with down-regulation of gene expression, but this association was not observed for *ANKRD46* (Figure 3c, d).

**Figure 4:**
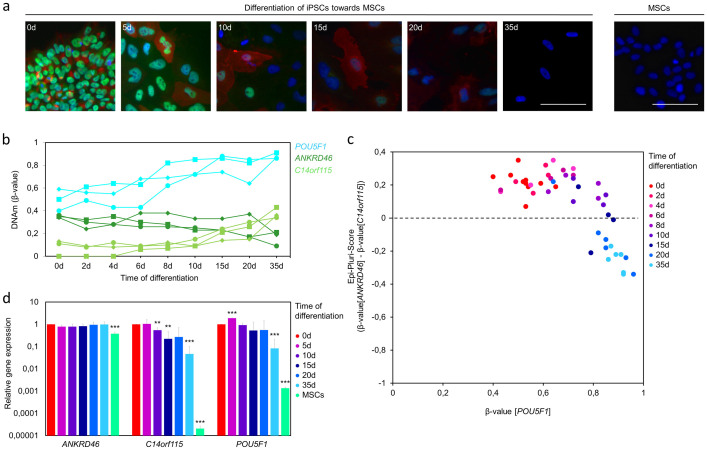
Analysis of pluripotency markers during differentiation of iPSCs towards MSCs. (a) Immunofluorescence analysis of TRA-1-60 (red) and OCT4 (green) in iPSCs and in the course of differentiation towards iPSC-derived MSCs^31^ (nuclear counterstaining with DAPI: blue; scale bar = 100 μm). (b) Time course of DNAm levels in cg23737055 (*ANKRD46*), cg22247240 (*C14orf115*), and cg13083810 (*POU5F1*). Three different iPSC clones are indicated by different symbols. (c) Epi-Pluri-Score classification of iPSC-derived MSCs in the course of differentiation. DNAm levels in cg13083810 (*POU5F1*) discriminate early differentiation changes better – it is therefore useful to complement the Epi-Pluri-Score. (d) Quantitative RT-PCR analysis of *ANKRD46*, *C14orf115*, and *POU5F1* in the course of iPSC-differentiation towards MSCs. (n = 3; differential expression compared to iPSCs: * = P < 0.05; ** = P < 0.02; *** = P < 0.001).

Subsequently, we analyzed if the Epi-Pluri-Score facilitates also discrimination of iPSC-derived differentiated cells. To this end, we have differentiated iPSCs towards MSCs using our recently described protocol, which is based on differentiation in culture medium with human platelet lysate^31^. Immunofluorescence staining of OCT4 and TRA-1-60 demonstrated down-regulation within 10 to 20 days under these differentiation conditions (Figure 4a). Accordingly, pyrosequencing revealed hypermethylation within the CpG site of *POU5F1* after 8 days of differentiation, whereas DNAm changes in *C14orf115* and *ANKRD46* occurred rather after 15 and 20 days, respectively (Figure 4b). Therefore, reliable classification of differentiated cells by the Epi-Pluri-Score was only possible after 20 days of differentiation towards MSCs (Figure 4c)^31^. This relatively late response can be attributed to the differentiation protocol as also reflected by immunophenotypic kinetics and down-regulation of pluripotency-associated genes. In fact, gene expression of *POU5F1* and *C14orf115* was modestly down-regulated, which may correspond to the modest hypermethylation of the corresponding CpGs upon differentiation (Figure 4d).

Furthermore, we have exemplarily tested the Epi-Pluri-Score on early iPSCs because it has been suggested that low-passage iPSCs retain a transcriptional memory of the original cells^32^. Our iPSCs were clearly classified as pluripotent at 65 days after reprogramming with episomal plasmids and hence the procedure facilitates analysis of early iPSC clones (Supplemental Figure S6).

## Discussion

High-throughput production of iPSCs needs to be complemented by reliable methods for high-throughput analysis of potentially reprogrammed clones^33^. In this study, we describe three CpGs that facilitate fast and cost-effective evaluation of pluripotency. Our classifier was validated on a very large set of DNAm profiles and this proved high sensitivity and specificity.

In fact, a similar degree of discrimination between ESCs/iPSCs and non-pluripotent cells can be achieved by quantitative gene expression analysis of a small panel of pluripotency-associated genes (as depicted for *POU5F1* in Supplemental Fig. S3c). However, DNAm has several important advantages over gene expression as biomarker: 1) In contrast to RNA, DNA is more stable and can be isolated from cell pellets or shipped at room temperature to an external service provider; 2) β-values provide quantitative measures that can be easily addressed without definition of cutoffs or normalization to reference genes; and 3) the state of cellular differentiation is directly reflected by epigenetics and thus DNAm changes hold valuable information for cell fate decisions. Many other studies demonstrated that the DNAm patterns change dramatically during reprogramming into iPSCs indicating that it is possible to use DNAm profiles for classification of pluripotent and non-pluripotent cells^13^,^15^. A predictor that uses many CpGs might be more robust – this certainly is one of the strengths of the PluriTest analysis based on gene expression profiles^11^ – but analysis by microarray or deep sequencing technology is relatively expensive and requires complex bioinformatics. In contrast, our Epi-Pluri-Score is very cost-effective and simple. Furthermore, pyrosequencing can be performed with very small amounts of bisulfite converted DNA (even below 100 ng).

In theory, combination of more relevant CpGs strengthens the predictive power. However, in this study the high accuracy of the Epi-Pluri-Score was not further increased by combination of 68 CpGs and analysis of these additional CpGs by pyrosequencing would raise the costs significantly. We have recently described senescence-associated DNAm changes, which are continuously acquired during culture expansion of primary cells, and which are almost entirely reversed in iPSCs^34^,^35^. These senescence-associated DNAm changes are gradually recapitulated upon *in vitro* differentiation of pluripotent cells^31^. In analogy, age-associated DNAm changes accumulate continuously during aging of the organism and are reversed by reprogramming into iPSCs^36^,^37^. Senescence- and age-associated DNAm changes may complement quality control of iPSCs. However, these epigenetic signatures were overall less efficient to separate somatic cells from pluripotent cells.

Correct classification of partially reprogrammed cells – which have been attributed as being positive for one pluripotency marker while being negative for others^8^,^27^ – is a particular challenge for pluripotency assays. Our Epi-Pluri-Score classified one partially reprogrammed cell line as non-pluripotent. We were also able to identify seemingly improper reprogrammed cells and misarranged GEO submissions with our analysis. However, further research is required to estimate reliability of the Epi-Pluri-Score on partially reprogrammed cells. The same applies to DNAm of the *POU5F1*-related CpG in embryonal carcinomas and to the “Parthenogenic-Score”. The bottleneck is that, so far, relatively few datasets for such thoroughly characterized cell lines are publically available. For a pluripotency marker it is also critical to discern early events of differentiation and to assess uniformity of iPSCs and ESCs. Particularly on the later issue the availability of reliable biological data is scarce and therefore efficiency of our Epi-Pluri-Score could not be tested.

Overall, DNAm changes in the course of differentiation do not seem to occur very rapidly. Particularly DNAm within *POU5F1* turned out to be helpful for identification of early differentiation events which may be due to the function of OCT4 as master regulator and gatekeeper of pluripotency^25^,^26^. Immunofluorescence staining of OCT4 and TRA-1-60 revealed similar kinetics as the Epi-Pluri-Score in the course of differentiation of iPSCs towards MSCs. However, immunofluorescence staining results are highly dependent on the staining procedure, fluorescence microscopic settings, and image section. In contrast, Epi-Pluri-Score analysis provides more standardized and quantitative results.

It may be important to determine whether or not iPSCs are primed to differentiate towards specific lineages. Expression of cell-line-specific outlier genes were reported to facilitate prediction of such bias, particularly if combined with functional differentiation assays^9^,^38^. On the other hand, iPSCs that perform poorly under directed differentiation of embryoid bodies may provide better results under other differentiation assays^9^,^33^,^38^. Our Epi-Pluri-Score is not suitable to discern lineage-specific bias – such molecular analysis is, to our knowledge, so far only possible by analysis of pluripotent cells (i.e. by qRT-PCR analysis using the Sorecard panel of relevant genes) in combination with their terminally differentiated states, via either spontaneous differentiation or directed differentiation into specific lineages^9^,^10^. In contrast, our method is rather a classifier for extreme states without power for quality control of lineage-specific differentiation potential. The Epi-Pluri-Score does not discriminate between iPSCs and ESCs. Furthermore, the Epi-Pluri-Score does not distinguish between normal pluripotent and parthenogenic ESCs, embryonal carcinomas, or other close derivatives with restricted differentiation potential. iPSCs may also harbor acquired mutations in key developmental genes or a specific genetic background. Thus, the Epi-Pluri-Score provides a minimum criterion for pluripotency assessment and for the above mentioned specific questions other approaches need to be considered^6^.

In summary, our Epi-Pluri-Score provides a simple and robust approach to support classification of pluripotent and non-pluripotent cells. It is based on DNAm at only three CpGs and therefore facilitates screening of multiple iPSC clones for quality control in a time-saving and cost-effective manner. However, for additional information – such as propensity of differentiation towards specific lineages – the method needs to be complemented by other assays.

## Methods

### DNA methylation-datasets used in this study

As training-dataset to derive the Epi-Pluri-Score we used 258 DNAm profiles, which were retrieved from the NCBI Gene Expression Omnibus (GEO) database (series numbers: GSE29290, GSE30870, GSE31848, GSE37066, and GSE40909; Supplemental Table S1). All of these DNAm profiles were generated on the Illumina HumanMethylation450 BeadChip^22^. Raw data were transformed into β-values, and manually classified as pluripotent, somatic, or *in vitro* differentiated based on the sample description on the GEO website as well as additional information in accompanying publications (pESCs and ECs were excluded for derivation of the Epi-Pluri-Score). Cells considered as pluripotent are ESCs or iPSCs and were reported to be tested for their pluripotency by teratoma formation or PluriTest analysis as well as by staining of typical pluripotency markers in the respective publications.

For rigorous validation of the Epi-Pluri-Score we used an independent dataset with 2,216 samples, which were analyzed on the Illumina HumanMethylation27 BeadChip (GEO series numbers: GSE24676, GSE25047, GSE25083, GSE25089, GSE25538, GSE26033, GSE26126, GSE26519, GSE26543, GSE26683, GSE27130, GSE29661, GSE29871, GSE30090, GSE30456, GSE30601, GSE30653, GSE30759, GSE32393, GSE32861, GSE32866, GSE34035, GSE34869, GSE36812, GSE36829, GSE40097, and GSE42646; Supplemental Table S2). DNAm profiles of parthenogenic ESCs, embryonic carcinomas, and teratocarcinomas were initially excluded.

### Derivation of the Epi-Pluri-Score

We focused on 25,978 CpGs that are present on both Illumina HumanMethylation BeadChip platforms (450 k and 27 k). Initially, each of these CpGs was tested on accuracy to classify samples of the training-dataset into pluripotent and non-pluripotent cells. In fact, many CpGs revealed a perfect accuracy and therefore we selected those CpG-sites with the maximum margin between pluripotent and somatic samples as a first criterion (Figure 1b). This criterion highlighted the CpG site cg22247240 (*C14orf115*), which is hardly ever methylated in pluripotent cells but methylated in non-pluripotent cells. The CpG-site cg23737055 (*ANKRD46*) was selected by using an additional criterion based on the classification of 18 samples at different stages of *in-vitro* differentiated pluripotent cells. Based on the DNAm levels at these two CpG-sites, the Epi-Pluri-Score is defined as: Epi-Pluri-Score = β-value [cg23737055] − β-value [cg22247240].

### Derivation of the Parthenogenic-Score

The Parthenogenic-Score was derived according to the first criterion mentioned above comparing six pESCs with 63 ESC/iPSC. This resulted in the formula: Parthenogenic-Score = β-value [cg18506672] − β-value [cg17769238] – 0.2. The subtraction of 0.2 was used to shift the cutoff value for classification to zero. Please note that this score has so far not been validated on independent datasets.

### Gene expression data and PluriTest analysis

Several studies of the training- and validation-datasets included gene expression data. We downloaded all of the gene expression profiles that have been analyzed on Illumina HumanHT-12 v3 or v4 microarrays. Raw data was quantile normalized together. PluriTest analysis was performed as previously described^11^. In addition, we used our previously published gene expression profiles of MSCs (GSE46019)^30^, iPSCs (GSE38806)^18^ and iPS-MSCs (GSE54766)^31^ to estimate expression levels of specific genes based on signal intensity of RMA normalized raw data.

### Cells used in this study

Mesenchymal stromal cells (MSCs) from bone marrow (BM)^18^, MSCs from adipose tissue (AT)^39^, MSCs from cord blood (CB), and HDFs^40^ were isolated after written consent using guidelines approved by the Ethic Committee on the Use of Human Subjects at the University of Aachen (Permit numbers EK128/09; EK187/08; EK163/07; and EK187/08, respectively). All other primary cells were taken after written consent according to the “Biobank” rules of the medical faculty of the University of Aachen (Permit Number: EK206/09 and EK127/12). Work with human ESCs has been approved by the Robert Koch Institute, Berlin, Germany (permit no. Az 1710-79-1-4-79). Reprogramming of HDFs with episomal plasmids was performed as described in detail before^41^. All other cell lines have been purchased (ATCC, Manassas, USA; Supplemental Table S3).

### Pyrosequencing

Genomic DNA was isolated using the NucleoSpin Tissue Kit (Macherey-Nagel, Düren, Germany) and 1 μg of DNA was sodium bisulfite-modified using the EZ DNA Methylation Kit (Zymo Research, Irvine, USA). The region of interest was amplified by PCR using the first primer pairs (Supplemental Table S4). A single strand linear DNA was prepared from the PCR product with the PyroMark Q96 Vacuum Prep Workstation (Qiagen, Hilden, Germany). The sequencing reaction was then performed with a gene specific sequencing primer on a PyroMark Q96 ID System and analyzed with the PyroMark CpG SW 1.0 software (Qiagen).

### Immunofluorescence staining

Immunofluorescence staining was performed on vitronectin (iPSCs) or gelatine-coated coverslips as described before^41^ using antibodies for TRA-1-60 (clone: TRA-1-60; Millipore, Darmstadt, Germany) and OCT3/4 (clone C-10; Santa Cruz Biotechnology, Dallas, USA). Images were taken on an Axioplan 2 fluorescence microscope (Carl Zeiss, Oberkochen, Germany).

### Quantitative RT-PCR

Total RNA was reverse transcribed into cDNA using the High Capacity Kit (Applied Biosystems, Carlsbad, USA). Semiquantitative RT-PCR was always performed in triplicates on an Applied Biosystems StepOnePlus device with Taqman Gene Expression Assays for *ANKRD46* (Hs01569215_m1), *C14orf115* (Hs00217248_m1), and *POU5F1* (Hs00999632_g1; all Life Technologies, Darmstadt, Germany). *GAPDH* (Hs02758991_g1; Life Technologies) was used as reference gene for normalization.

## Author Contributions

M.L., R.G., An. S. and W.W. made conception and design for this study; M.L. and Ar. S. performed bioinformatics; R.G. and C.S. performed pyrosequencing analysis; J.V., H.H., S.K. and M.Z. provided cells and assisted cell culture experiments. M.L., R.G. and W.W. wrote the main manuscript. All authors reviewed the manuscript.

## Supplementary Material

Supplementary Information Supplementary Information

Supplementary Information Supplementary Table S1

Supplementary Information Supplementary Table S2
