# Supplementary material for: Epigenetic Biomarker to Support Classification into Pluripotent and Non-Pluripotent Cells
Source: Sci Rep. 2015 Mar 10;5:8973. doi: 10.1038/srep08973 (PMC4354028; doi:10.1038/srep08973)
Supplement: Supplementary Information [file srep08973-s1.pdf]

SUPPLEMENTAL MATERIAL

Epigenetic Biomarker to Support Classification into Pluripotent and Non-Pluripotent Cells

Michael Lenz, Roman Goetzke, Arne Schenk, Claudia Schubert, Jürgen Veeck, Hatim Hemeda, Steffen Koschmieder, Martin Zenke, Andreas Schuppert, and Wolfgang Wagner

Index

Suppl. Fig. S1. DNAm profiles of *ANKRD46*, *C14orf115*, and *POU5F1* 2

Suppl. Fig. S2. Accuracy of individual CpGs in the validation-dataset 3

Suppl. Fig. S3. Epi-Pluri-Score analysis reveals improper reprogramming 4

Suppl. Fig. S4. Analysis of embryonal carcinomas and parthenogenic ESCs 5

Suppl. Fig. S5. Sequence information for pyrosequencing assays 6

Suppl. Fig. S6. Analysis of iPSCs early after reprogramming 6

Suppl. Tab. S2. Validation-dataset of DNAm profiles 7

Suppl. Tab. S3. Cell preparations used for pyrosequencing 7

Suppl. Tab. S4. Primers for pyrosequencing 9

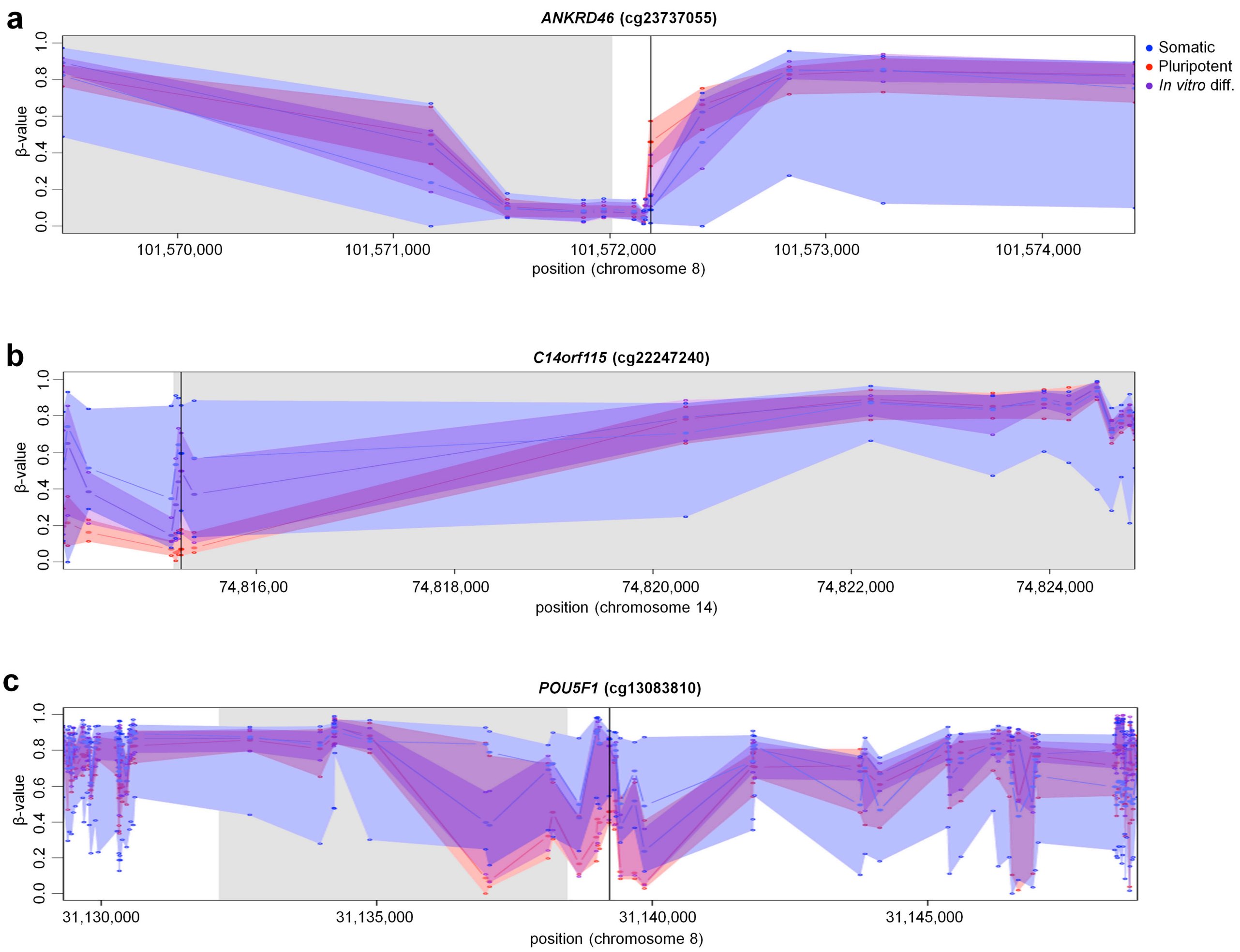

Suppl. Fig. S1. DNAm profiles of *ANKRD46*, *C14orf115*, and *POU5F1*

DNAm levels of CpGs on the Illumina HumanMethylation450 BeadChips corresponding to the genes *ANKRD46* **(a)**, *C14orf115* **(b)**, and *POU5F1* **(c)**. The gray background depicts the protein-coding region (Genome Reference Consortium human genome build 37). The vertical black line denotes location of the selected CpG for *ANKRD46* (TSS200; reverse strand), *C14orf115* (Exon 1), or *POU5F1* (TSS1500; reverse strand). The range (min. to max. β-value) of DNAm is presented for pluripotent (red), somatic (blue), and *in vitro* differentiated iPSC samples (violet). Particularly for *ANKRD46* pluripotency-associated hypermethylation is restricted to a relatively small genomic region.

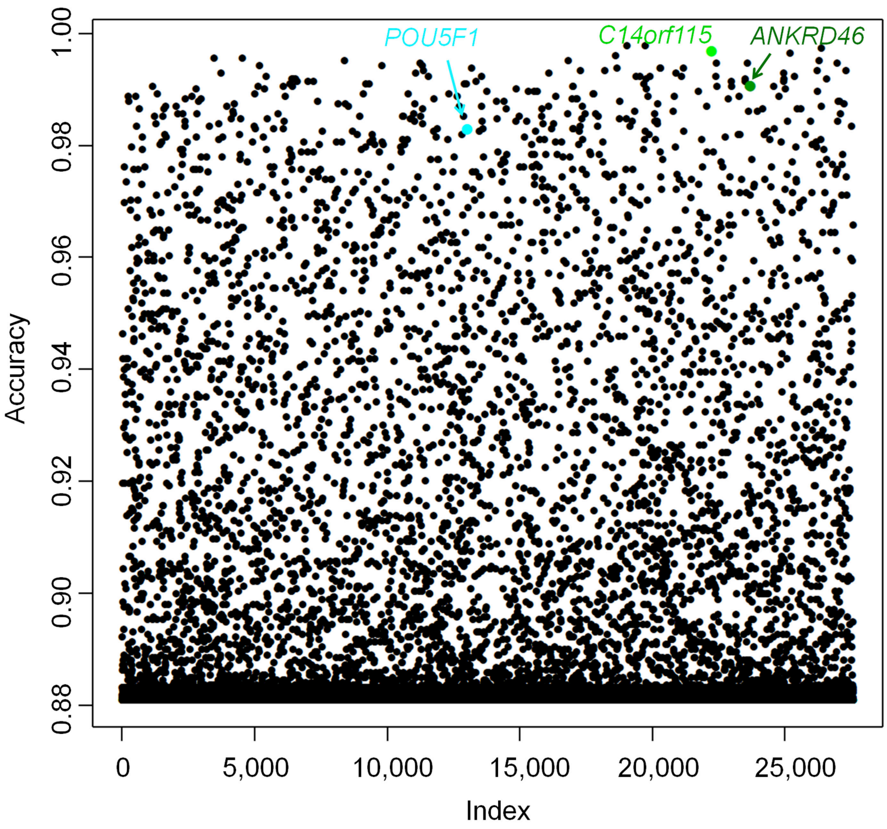

Suppl. Fig. S2. Accuracy of individual CpGs in the validation-dataset

For each of the CpG sites the accuracy to classify the samples of the validation-dataset (Illumina HumanMethylation27 BeadChip) into pluripotent and non-pluripotent cells was tested. The cutoff for classification was chosen in such a way that the accuracy is maximized for each CpG. Most CpG-sites are close to the baseline accuracy of 0.88 (i.e. the accuracy of classifying all samples as somatic). Particularly, the two CpG sites of the Epi-Pluri-Score (*C14orf115* and *ANKRD46*) are among the top performing CpGs.

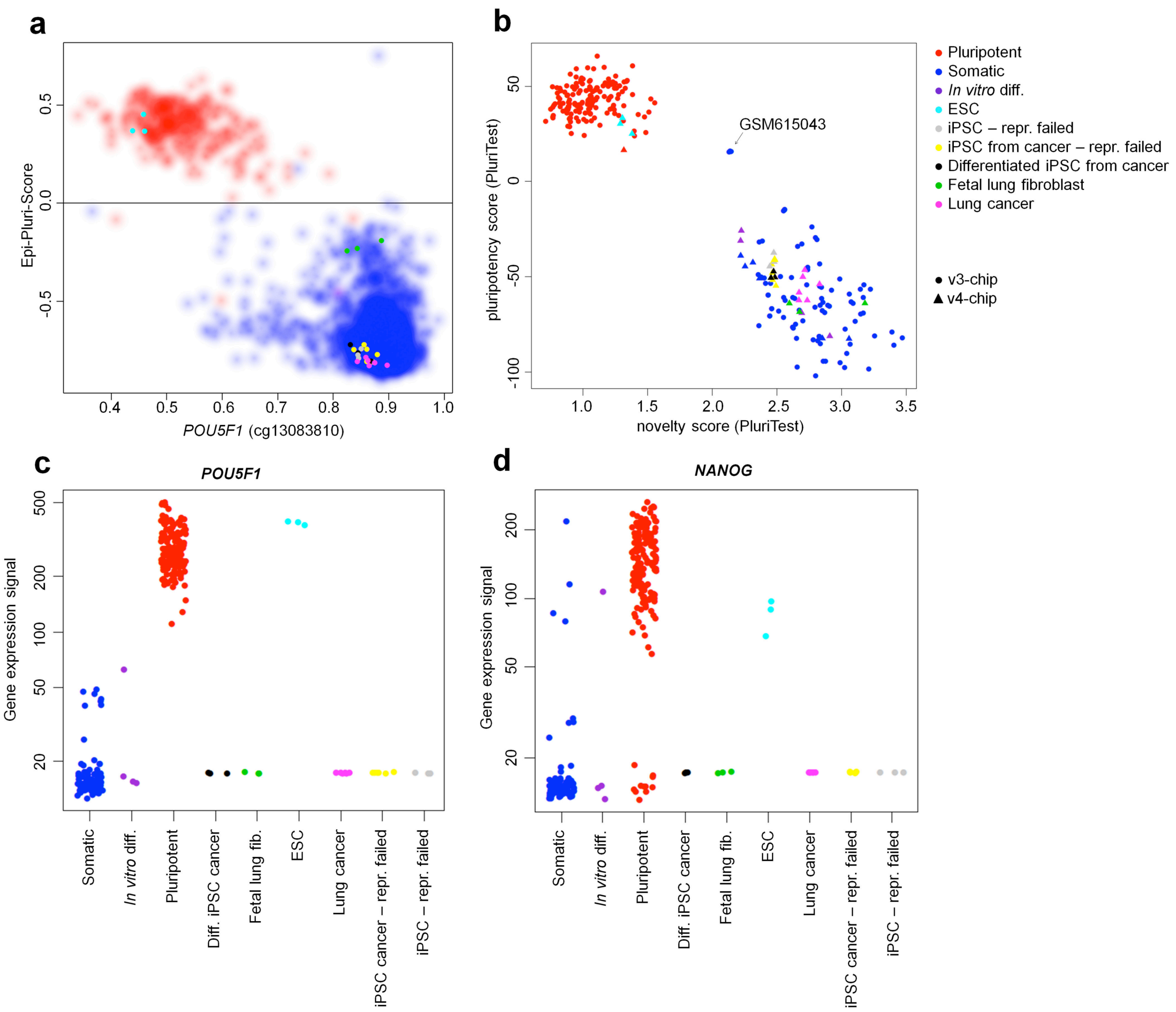

Suppl. Fig. S3. Epi-Pluri-Score analysis reveals improper reprogramming

(a) Our Epi-Pluri-Score was used on a dataset with “iPSCs” that – on first sight – appeared to be misclassified as non-pluripotent (GSE35912; gray and yellow points)[2]. Red and blue shaded areas reflect the reference data from Figure 2b (repr. = reprogramming, diff. = differentiation, fib. = fibroblast). **(b)** PluriTest analysis[1] of accompanying gene expression data confirm improper reprogramming of the “iPSCs” from this dataset (GSE35912; grey and yellow spots)[2]. The symbols reflect different Illumina HumanHT-12 microarray platforms (v3 = circles; v4 = triangles). Lack of reprogramming is further supported by exemplary analysis of the pluripotency associated genes **(c)** *POU5F1* and **(d)** *NANOG.* Thus, Epi-Pluri-Score analysis identified samples that rather seem to have wrong designation as pluripotent cells.

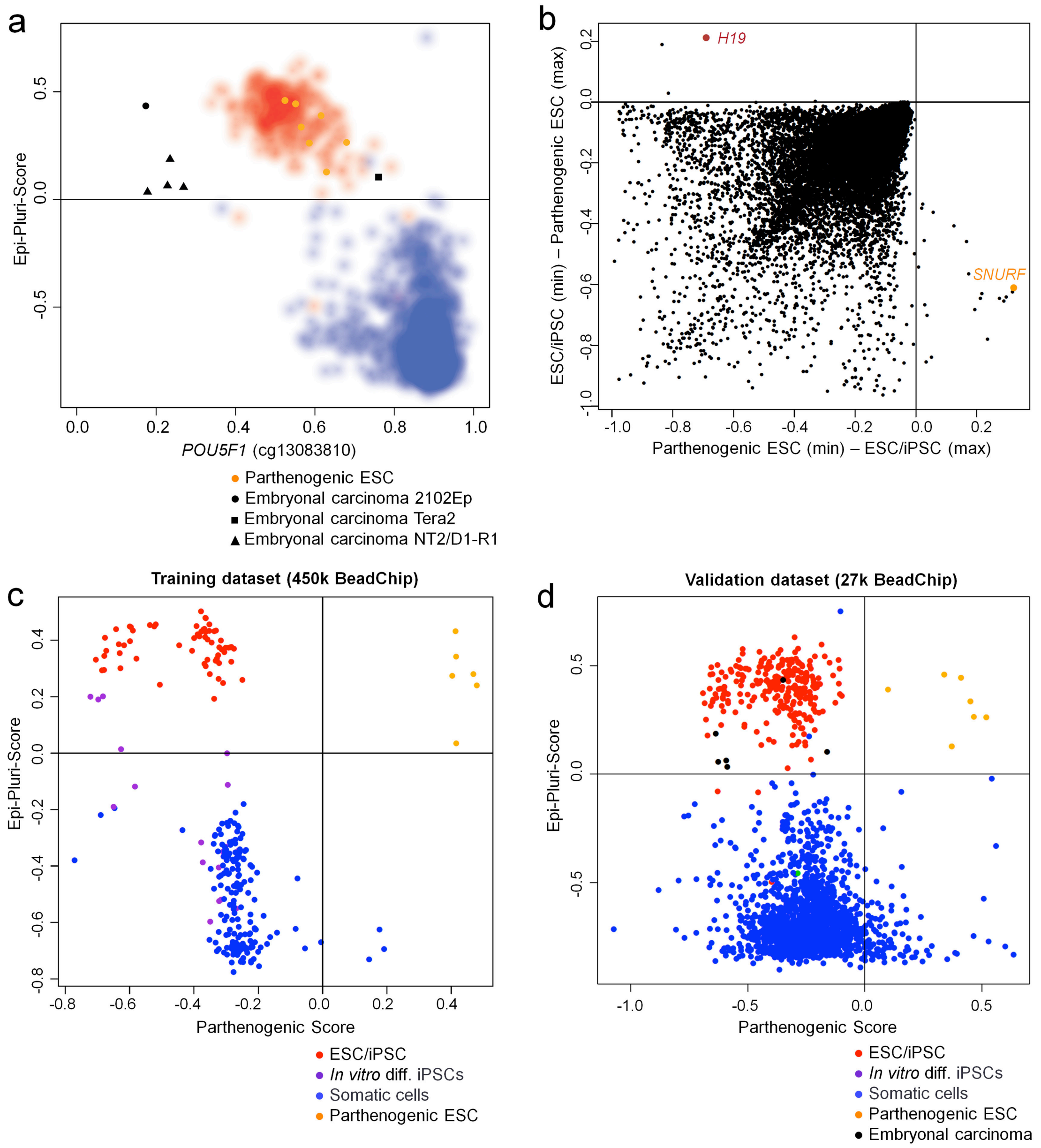

Suppl. Fig. S4. Analysis of embryonal carcinomas and parthenogenic ESCs

(a) The Epi-Pluri-Score is not able to distinguish between ESCs or iPSCs and embryonal carcinoma cell lines (ECs) or parthenogenic ESCs (pESCs). However, the CpG associated to *POU5F1* shows aberrant high or low DNAm for EC lines. These findings deserve further validation in independent datasets. **(b)** We selected two CpG sites for distinction of ESC/iPSC and pESC based on maximization of the margin between these two groups in the training-dataset. This is similar to the 1st criterion in the derivation of the Epi-Pluri-Score. Notably, genes associated with the best performing CpGs, i.e. *H19* and *SNURF*, are both related to imprinting. **(c)** A “Parthenogenic Score” was derived by combination of these two CpGs to distinguish ESCs/iPSCs and pESCs on the training-dataset. **(d)** The same score is also able to distinguish these pESCs in the validation-dataset. However, it has to be noted that the pESC cell lines in the validation-dataset are exactly the same as in the training-dataset (with additional technical replica).

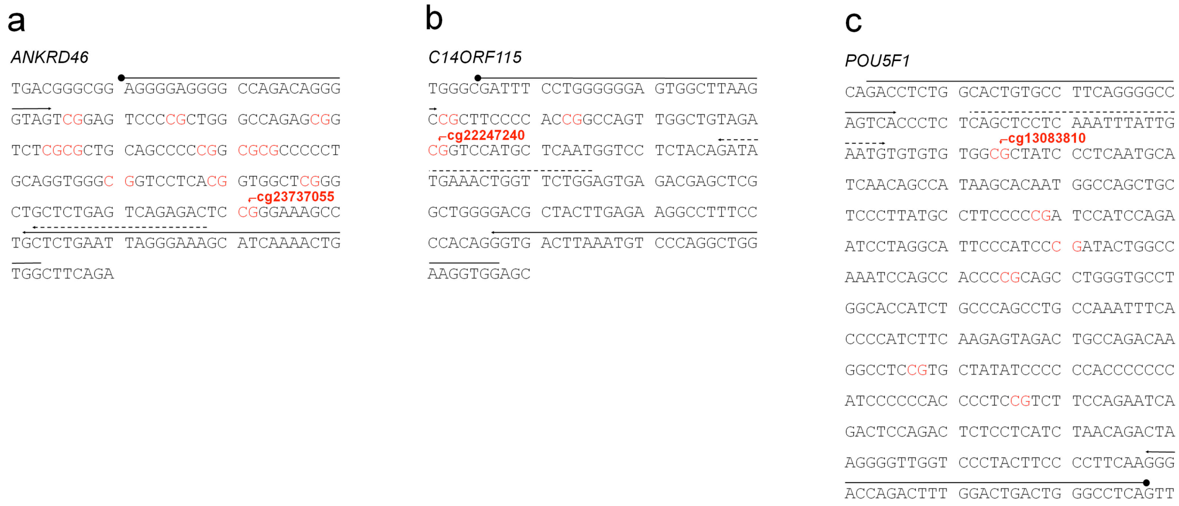

Suppl. Fig. S5. Sequence information for pyrosequencing assays

Sequences covered by the pyrosequencing assays are demonstrated for **(a)** *ANKRD46*, **(b)** *C14orf115*, and **(c)** *POU5F1*. Solid lines depict hybridization sites of forward primers and reverse primers (● = biotinylated) for PCR amplification. Position of the sequencing primer is demonstrated as a broken line. The corresponding CpG sites of the Illumina HumanMethylation BeadChip arrays are highlighted (cg23737055, cg22247240, and cg13083810).

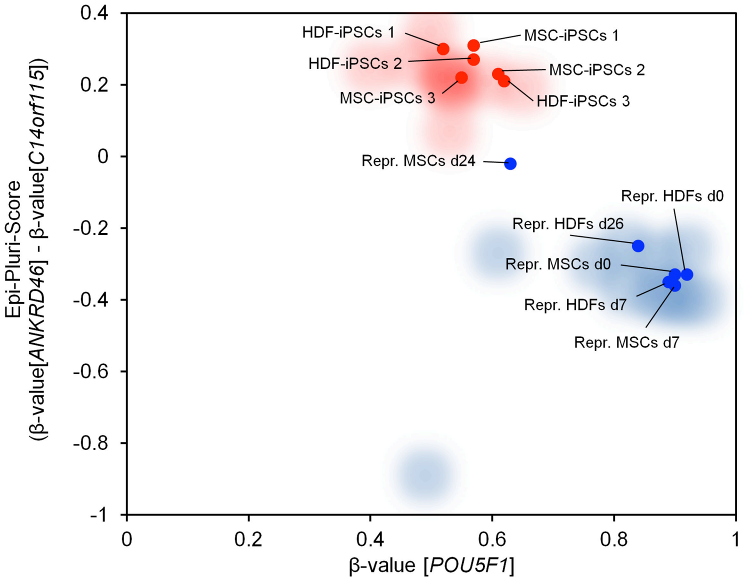

Suppl. Fig. S6. Analysis of iPSCs early after reprogramming

Human dermal fibroblasts and bone marrow mesenchymal stromal cells were reprogrammed by episomal plasmids (d0)[36]. After 7 days (first morphological changes) and after 24 to 26 days (picking of colonies) the cells were still associated with non-pluripotent cells, which may be attributed to the majority of MEF-feeder layer cells. Upon expansion of several clones under feeder-free conditions (d62 and d65 for reprogrammed BM MSCs and HDFs, respectively) they were clearly associated with pluripotent cells. Red and blue clouds refer to the pluripotent and fibroblast/MSC samples of figure 3b, respectively.

Suppl. Tab. S1. Training-dataset of DNAm profiles

This table comprises information on 258 samples (all 450k Illumina BeadChip platform) that were used as training-dataset to derive the Epi-Pluri-Score as well as 6 pESC samples. This table is provided as separate EXCEL file.

Suppl. Tab. S2. Validation-dataset of DNAm profiles

This table comprises information on 2.216 samples (all 27k Illumina BeadChip platform) that were used for validation of the Epi-Pluri-Score as well as 6 EC and 12 pESC samples. This table is provided as separate EXCEL file.

Suppl. Tab. S3. Cell preparations used for pyrosequencing

**Cell type**	**Characteristics**	**β-value [*ANKRD46*]**	**β-value [*C14orf115*]**	**β-value [*POU5F1*]**	**Epi-Pluri-Score**
iPSCs					
iPSC 3.11	derived from CD117+ BM cells; P49	0.35	0	0.50	0.35
iPSC 3.29	derived from CD117+ BM cells; P51	0.34	0.13	0.59	0.21
iPSC 3.40	derived from CD117+ BM cells; P48	0.36	0.11	0.40	0.25
M2C6	iPSCs derived from BM MSCs	0.30	0.04	0.47	0.26
M4C4	iPSCs derived from BM MSCs	0.21	0	0.53	0.21
M5C1	iPSCs derived from BM MSCs	0.23	0	0.53	0.23
Mastocytosis117	iPSCs from mastocytosis patient	0.30	0.11	0.54	0.19
Mastocytosis73	iPSCs from mastocytosis patient	0.26	0.07	0.65	0.19
Mastocytosis23	iPSCs from mastocytosis patient	0.32	0.10	0.52	0.22
H9 iPSC hybrid	iPSCs hybridized with H9 ESCs	0.13	0.06	0.53	0.07
HDF-iPSCs 1	iPSCs from HDFs (65d of repr.)	0.30	0	0.52	0.30
HDF-iPSCs 2	iPSCs from HDFs (65d of repr.)	0.27	0	0.57	0.27
HDF-iPSCs 3	iPSCs from HDFs (65d of repr.)	0.29	0.08	0.62	0.21
MSC-iPSCs 1	iPSCs from BM MSCs (62d of repr.)	0.31	0	0.57	0.31
MSC-iPSCs 2	iPSCs from BM MSCs (62d of repr.)	0.23	0	0.61	0.23
MSC-iPSCs 3	iPSCs from BM MSCs (62d of repr.)	0.28	0.06	0.55	0.22
ESCs					
HES2	Human embryonic stem cell line	0.29	0	0.45	0.29
HES3	Human embryonic stem cell line	0.29	0.08	0.19	0.21
Differentiated iPSCs					
iPSC 3.11 d2	Differentiated for 2d in HPL-medium	0.32	0	0.61	0.32
iPSC 3.29 d2	Differentiated for 2d in HPL-medium	0.24	0.09	0.56	0.15
iPSC 3.40 d2	Differentiated for 2d in HPL-medium	0.30	0.08	0.49	0.22
M2C6 d2	Differentiated for 2d in HPL-medium	0.32	0.06	0.62	0.26
iPSC 3.11 d4	Differentiated for 4d in HPL-medium	0.35	0	0.64	0.35
iPSC 3.29 d4	Differentiated for 4d in HPL-medium	0.28	0.08	0.55	0.20
iPSC 3.40 d4	Differentiated for 4d in HPL-medium	0.28	0.12	0.43	0.16
M2C6 d4	Differentiated for 4d in HPL-medium	0.35	0.05	0.72	0.30
iPSC 3.11 d6	Differentiated for 6d in HPL-medium	0.30	0.06	0.63	0.24
iPSC 3.29 d6	Differentiated for 6d in HPL-medium	0.38	0.09	0.68	0.29
iPSC 3.40 d6	Differentiated for 6d in HPL-medium	0.26	0.09	0.43	0.17
M2C6 d6	Differentiated for 6d in HPL-medium	0.34	0.08	0.72	0.26
iPSC 3.11 d8	Differentiated for 8d in HPL-medium	0.28	0.07	0.82	0.21
iPSC 3.29 d8	Differentiated for 8d in HPL-medium	0.38	0.12	0.69	0.26
iPSC 3.40 d8	Differentiated for 8d in HPL-medium	0.26	0.10	0.62	0.16

M2C6 d8	Differentiated for 8d in HPL-medium	0.28	0.16	0.82	0.12
iPSC 3.11 d10	Differentiated for 10d in HPL-medium	0.23	0.09	0.85	0.14
iPSC 3.29 d10	Differentiated for 10d in HPL-medium	0.33	0.09	0.72	0.24
iPSC 3.40 d10	Differentiated for 10d in HPL-medium	0.25	0.15	0.72	0.10
M2C6 d10	Differentiated for 10d in HPL-medium	0.26	0.18	0.84	0.08
iPSC 3.11 d15	Differentiated for 15d in HPL-medium	0.23	0.21	0.86	0.02
iPSC 3.29 d15	Differentiated for 15d in HPL-medium	0.33	0.14	0.74	0.19
iPSC 3.40 d15	Differentiated for 15d in HPL-medium	0.23	0.24	0.88	-0.01
M2C6 d15	Differentiated for 15d in HPL-medium	0.03	0.24	0.79	-0.21
iPSC 3.11 d20	Differentiated for 20d in HPL-medium	0.17	0.26	0.82	-0.09
iPSC 3.29 d20	Differentiated for 20d in HPL-medium	0.37	0.15	0.64	0.22
iPSC 3.40 d20	Differentiated for 20d in HPL-medium	0.17	0.30	0.85	-0.13
M2C6 d20	Differentiated for 20d in HPL-medium	0.03	0.21	0.85	-0.18
M4C4 d20	Differentiated for 20d in HPL-medium	0.03	0.37	0.96	-0.34
M5C1 d20	Differentiated for 20d in HPL-medium	0.07	0.31	0.93	-0.24
iPSC 3.11 d35	Differentiated for 35d in HPL-medium	0.21	0.43	0.91	-0.22
iPSC 3.29 d35	Differentiated for 35d in HPL-medium	0.19	0.36	0.87	-0.17
iPSC 3.40 d35	Differentiated for 35d in HPL-medium	0.09	0.34	0.86	-0.25
M2C6 d35	Differentiated for 35d in HPL-medium	0.03	0.25	0.89	-0.22
M4C4 d35	Differentiated for 35d in HPL-medium	0.04	0.38	0.92	-0.34
M5C1 d35	Differentiated for 35d in HPL-medium	0.06	0.39	0.92	-0.33
Fibroblasts and MSCs					
HDFs P0	Cultured in HPL-Medium	0	0.25	0.82	-0.25
HDFs P3	Cultured in HPL-Medium	0	0.28	0.87	-0.28
HDFs P11	Cultured in HPL-Medium	0	0.30	0.77	-0.30
HDFs P16	Cultured in HPL-Medium	0	0.27	0.61	-0.27
Repr. HDFs d0	HDFs; P2; Cultured in HPL-Medium	0.01	0.34	0.92	-0.33
Repr. HDFs d7	HDFs; 7d of repr.	0	0.35	0.89	-0.35
Repr. HDFs d26	HDFs; 26d of repr.	0.04	0.29	0.84	-0.25
Repr. MSCs d0	BM MSCs; P2; in HPL-Medium	0.01	0.34	0.90	-0.33
Repr. MSCs d7	BM MSCs; 7d of repr.	0.02	0.38	0.90	-0.36
Repr. MSCs d24	BM MSCs; 24d of repr.	0.18	0.20	0.63	-0.02
CB MSCs 1	Isolated from cord blood	0.03	0.43	0.94	-0.40
CB MSCs 2	Isolated from cord blood	0.03	0.40	0.82	-0.37
AT MSCs 23	Cultured in HPL-medium; P1	0.04	0.93	0.49	-0.89
AT MSCs 27	Cultured in FCS; P1	0.02	0.41	0.87	-0.39
BM MSCs 102	Cultured in HPL-Medium	0.01	0.27	0.92	-0.26
BM MSCs 105	Cultured in HPL-Medium	0.01	0.41	0.91	-0.40
BM MSCs 106	Cultured in HPL-Medium	0.01	0.39	0.89	-0.38
Tumor and non-tumor cell lines					
BT-20	Mamma CA	0	0.83	0.82	-0.83
BT-474	Mamma CA	0.04	0.84	0.88	-0.80
UACC-3199	Mamma CA	0	0.72	0.88	-0.72
T-47D	Mamma CA	0.06	0.85	0.85	-0.79
MDA-MB-231	Mamma CA	0.01	0.92	0.68	-0.91
MDA-MB-436	Mamma CA	0.01	0.88	0.84	-0.87
MCF-7	Mamma CA	0.05	0.85	0.90	-0.80
MCF-10A	Normal breast epithelium	0.03	0.83	0.90	-0.80
MCF-12A	Normal breast epithelium	0.03	0.83	0.88	-0.80
ZR-75-1	Mamma CA	0.02	0.82	0.89	-0.80
J82	Bladder CA	0.02	0.87	0.82	-0.85
HCV29	Normal urothelium	0.04	0.79	0.87	-0.75
RT4	Urinary Bladder PA	0.03	0.81	0.89	-0.78
HL60	Human promyelocytic leukemia cells	0.07	0.78	0.83	-0.71

DU145	Prostate CA	0.07	0.76	0.93	-0.69
PC-3	Prostate CA	0.06	0.79	0.89	-0.73
HaCaT	Immortalized human keratinocytes	0.03	0.77	0.93	-0.74
Primary cells					
HUVECs	Endothelia; umbilical cord; P3	0.01	0.42	0.94	-0.41
HOMCs	Isolated from omentum majus; P0	0.01	0.71	0.93	-0.70
DPSCs	Isolated from dental pulp; P6	0.02	0.80	0.95	-0.78

Legend for Suppl. Tab. S3: CD117+ BM cells = hematopoietic progenitor cells from bone marrow; BM = bone marrow; P = passage; HDF = human dermal fibroblast; ESC = embryonic stem cell; CB = cord blood; FCS = fetal calf serum; HPL = human platelet lysate; CA = carcinoma; PA = papilloma; HOMC = human omentum majus cell; HUVEC = human umbilical vein endothelial cell; DPSC = dental pulp stem cell; AT = adipose tissue; repr. = reprogramming.

The Epi-Pluri-Score is defined as β-value [*ANKRD46*] – β-value [*C14orf115*].

Suppl. Tab. S4. Primers for pyrosequencing

Primer	CpG-ID	Sequence
ANKRD46-seq	ANKRD46 (cg23737055)	5´-CACAATTTTAATACTTTCCC-3´
ANKRD46-for		5´-Biotin-AGGGGAGGGGTTAGATAGGGGTAG-3´
ANKRD46-rev		5´-CCACAATTTTAATACTTTCCCTAATTCAAAAC-3´
C14ORF115-seq	C14ORF115 (cg22247240)	5´-CCAAAACCAATTTCATATC-3´
C14ORF115-for		5´-Biotin-GATTTTTTGGGGGGAGTGGTTTAAGT-3´
C14ORF115-rev		5´-CCACCTTCCAACCTAAAACATTTAAATCACC-3´
POU5F1/OCT4-seq	POU5F1/OCT4 (cg13083810)	5´-TAGTTTTTTAAATTTATTGAATG-3´
POU5F1/OCT4-for		5´-GATTTTTGGTATTGTGTTTTTAGGGGTTAGTTA-3´
POU5F1/OCT4-rev		5´-Biotin-TAAAACCCAATCAATCCAAAATCTAATCCC-3´

Reference List

1. Muller.F.J. *et al.* A bioinformatic assay for pluripotency in human cells. *Nat. Methods* **8**. 315-317 (2011).

2. Mahalingam.D. *et al.* Reversal of aberrant cancer methylome and transcriptome upon direct reprogramming of lung cancer cells. *Sci. Rep.* **2**. 592 (2012).
